# Problems with the accurate reporting of COVID-19 statistics in Iran 

**Published:** 2020

**Authors:** Freshteh Osmani

**Affiliations:** 1 *Dentistry Clinical Research Development Center, Birjand University of Medical Sciences, Birjand, Iran*; 2 *Infection Disease Research Center, Birjand University of Medical Sciences, Birjand, Iran *

 Coronaviruses are a large group of viruses ranging from the common cold to severe acute respiratory syndrome (SARS). Every day, the number of countries affected by the coronavirus increases, and as the virus progresses it will likely become a pandemic. Given the increasing numbers of infected cases and deaths, the coronavirus is becoming a challenging problem worldwide (1, 2). The epidemic of COVID-19 started from Wuhan, China, in late 2019 and spread to other countries. At the time of writing (June 15, 2020), 7,995,877 people in about 200 countries were infected, and 435,598 had died of this coronavirus disease (3).World statistics on COVID-19 show that the outbreak and mortality rate of this disease is much higher than that of the Influenza A or SARS-CoV viruses (4).

Given the growing importance of this issue, the WHO has published information on confirmed, recovered, and death cases of coronavirus, which is available on RamiKrispin dataset (3) and updated daily. As can be seen, newly confirmed, recovered, and death cases are recorded daily (5). Actually, every newly confirmed case, after a while, either recovers or dies.

Except for China, three countries (Italy, Iran, and South Korea) have had the highest numbers of confirmed cases and deaths from the coronavirus with much variation from other countries (especially in Italy and Iran, up to March 15). These countries have also had the highest numbers of days of involvement with the virus compared to most other countries. The differences in the number of deaths could be due to the large number of confirmations that could be considered an indirect indicator of the heavier healthcare burden on these countries (5). This explanation was proposed by the study of Yu et al. (6). 

In the following, some problems with the lack of accurate statistics for this serious public health threat are discussed.

**Figure 1:**
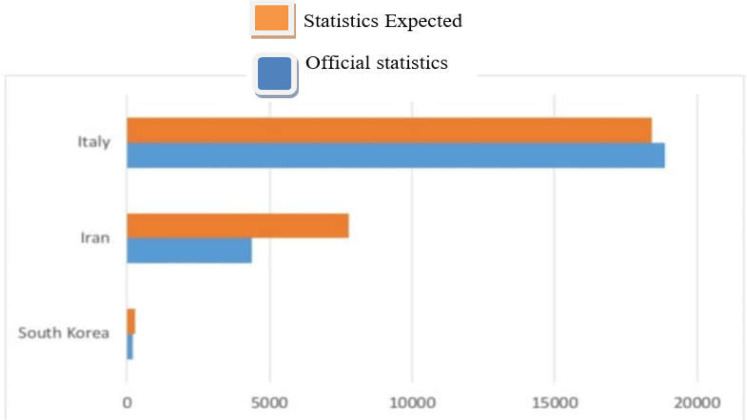
Official reported statistics and the expected number of deaths in a thirty-day period in 3 countries (5)

A recent article reported the expected number of deaths in a thirty-day period for three countries: Korea, Iran, and Italy. In this chart, the forecast numbers were shown in orange color, and the statistics officially reported by the three countries were recorded together in blue. It seems that the biggest difference between the official and estimated numbers is seen in the numbers from Iran (Figure 1) (5).

I believe that the officials of the Health Ministry honestly report all positive cases that end in death, but it would be better to report the statistics of deaths of people that were not tested for corona for any reason or were diagnosed based only on CT scans.

Another reason for the lower number of cases is the lack of accurate registration of statistics, especially in the early epidemic phase of this disease. Returning cases from medical records and re-registering the accurate statistics can solve this problem. 

The gap in the internal structure of the administration has severely disrupted management planning, and its financial resources are severely limited. Moreover, its foreign relations are in serious trouble. Other incidents, especially those occurring in recent months, have led to growing public distrust of this administration.

What happens when a doctor, based on his or her experience, believes that hospitalized patients have corona, but higher authorities only refer to cases that have tested positive? The result is that a province turns from a white state into a red one very quickly. 

Maybe, the reason for the high mortality rate of patients in Iran is that the number of patients is not accurate. There should be two statistics, the official ones and the estimated ones. Estimated statistics are important for people. The official statistics could be zero, as it was before the first death from COVID-19.

Furthermore, physicians' perceptions of patients as well as statistical estimates of the number of patients must be trusted, and all statistics and atlases of COVID-19 in Iran must be prepared and distributed based on statistical estimates. It is necessary to trust the physicians' diagnoses of the number of patients as well as statistical estimates. The efforts of the Health Ministry should be to bring official statistics closer to analytical and estimated ones. Ignoring the estimated statistics leads to no other result than deceiving ourselves.

The role of statistical science is to express many facts through estimation.

**Corona and its statistical ambiguities**

After the coronavirus entered Iran, the most important news became the daily statistics on the incidence, death, and recovery of patients with this virus in the country, provinces, cities, and even smaller urban areas.

Despite the willingness of health officials to be transparent in providing statistics, there are serious structural flaws and ambiguities in the method of information collection and reporting. Lack of attention to these ambiguities will lead to unrealistic figures, doubt in the accuracy of the statistics, confusion among the public, and disruption of evidence-based planning and decision-making.

The most important ambiguities regarding the collection, extraction, and reporting of coronavirus statistics are presented.

**Causes of the underestimation of COVID-19 mortality in Iran**

Initially, there were limitations in diagnosing, such as the lack of diagnosis tools, and relying solely on PCR results led to an obvious undercounting of patients. Thus, even WHO officials declared that the statistics were limited to one fourth of the real number of patients. This low number undermines the statistics. It is basically impossible to make policy based on statistics with serious shortcomings.

It is impossible to give an exact figure for coronavirus-related deaths, because the official toll reflects only the minority who are screened and test positive for the virus. “There are many people we don’t know are sick that could die at any point, and many people die from respiratory problems that can’t immediately be attributed to COVID-19.”

WHO experts agree that Iran’s infection rate is underestimated, a fact they say Iran’s leadership acknowledges. Iran isn’t alone in this; infection rates are underestimated almost everywhere the disease is present, particularly in countries where testing is hard to access unless a patient is hospitalized**.**

Another issue is related to the registration time. The results of people tested a few days before the results are announced will be included in the diagnostic day statistics. Analyses, forecasts, and positive rate increases are also based on the same false statistics. It is clear that this procedure leads to serious problems.

Also, people who test positive for COVID-19 and die some days later will have their death statistics recorded at the time of death, and this statistic will mislead the estimates and predictive statistical models.

Understanding the necessity of using registration statistics (recording and extracting statistics from routine executive processes) will compensate for backwardness in this area and create the conditions for the fast, inexpensive, and accurate collection of far–from-conflicting and unrealistic information. Therefore, this method should be considered.

**Problems related to data analysis**

The ability of experts to analyze information, estimates, comparisons, predictions, etc. is not sufficiently used, there is no coherent program for data analysis, and everyone uses his/her own model and method and reaches a unique analysis. It would be better for the statistics related to the patients, those who recover, and those who die from COVID-19 to be made available to the expert analysts in more detail so that the future of the country can be planned with more confidence and accuracy. 
